# Indo-Caribbean Youth and Suicidal Behavior: A Systematic Review

**DOI:** 10.3390/ijerph21060801

**Published:** 2024-06-19

**Authors:** Raul Ruiz Camacho, Shiryn D. Sukhram

**Affiliations:** Department of Biology, College of Staten Island, Staten Island, NY 10314, USA; raul.ruizcamacho@cix.csi.cuny.edu

**Keywords:** bereavement, Caribbean, suicide, youth, adolescence

## Abstract

The suicide rates in Guyana, Suriname and Trinidad and Tobago are among the highest in the Americas, containing significant Indo-Caribbean populations that are suggested to be most vulnerable to suicide. This systematic review analyzes the existing literature and identifies knowledge gaps in risk and protective factors against suicide in these countries. The literature search conducted followed PRISMA guidelines using the PubMed and APA PsycInfo databases. The PRISMA flow diagram illustrated that eight scholarly papers were eligible for inclusion. Included literature examined stratified data focused on the aforementioned countries, as well as their Indo-Caribbean adolescent populations. Excluded literature did not mention suicidality, adolescents, Indo-Caribbeans, or the focal countries or was focused on the Jonestown mass murder–suicide event. The studies encompassed 6581 individuals. Identified risk factors include social stigma regarding suicide, mental health resource scarcity, and difficult socioeconomic conditions. The identified protective factors for youth include religious/spiritual practices and group activities. Limitations include database quantity, risk of publication bias, and the small sample for each study. A prevailing social stigma regarding suicide was identified. Greater research is needed relating to effects of suicide legislation, bereavement experiences, sociocultural contexts, geography, migration patterns, and culturally compatible interventions to aid future suicide prevention efforts. The protocol was registered with PROSPERO (CRD42023417494).

## 1. Introduction

Suicide is a preventable cause of mortality, and while no single solution exists to reduce or eliminate suicidal behaviors, organized prevention efforts are crucial to reduce the number of lives lost. Suicidal behaviors include suicidal ideation (the thought of planning or attempting suicide), non-fatal suicide attempts (where an individual does not lose their life), and fatal suicide attempts (where an individual loses their life). Suicide mortality is a global public health concern, and it is far more pervasive in lower- to middle-income countries (LMICs) where access to necessary services or resources may be limited for people in the most susceptible communities [1].

In the Americas, suicide is noticeably prevalent within the countries of Guyana, Suriname, and Trinidad and Tobago. All these countries share a significant presence of Indo-descendant communities within their populations, who trace their origins to the indentured servants from British India who were taken to the British and Dutch colonial regions to provide cheap agricultural labor [2]. The respective crude suicide rates per population including both genders among all ages are 40.28 per 100,000, 25.37 per 100,000, and 8.68 per 100,000 according to 2023 World Health Organization (WHO) data [3]. When specifically observing suicide mortality rates for ages 15–19 years in these countries, a similar trend is identified that highlights the presence of systemic issues that reach adolescents as well. Guyana and Suriname hold the highest and second-highest suicide rates in the Americas, with Trinidad and Tobago holding the ninth-highest rate.

For the purposes of this review, the term “Indo-Caribbean” is used to describe the Indo-descendant ethnic groups in the countries of interest as a collective, as the three countries are members of the Caribbean Community (CARICOM) grouping of nations in the Caribbean region [4].

Studies from Guyana [5], Suriname [6], and Trinidad and Tobago [7] suggest that suicide is more prevalent among the Indo-Caribbean communities, who are the most susceptible to suicidal behavior due to socioeconomic factors. Risk factors such as the societal stigma attached to suicide and the insufficient mental health resources for individuals with suicidal ideation play a major role in the manifestation of suicidal behaviors. This review aims to examine the relationship between the aforementioned risk factors in Indo-Caribbean adolescents in Guyana, Suriname, and Trinidad and Tobago with suicidal behavior, as well as to identify gaps within the present literature to guide further research and eventual suicide prevention efforts. The PICO (population, intervention, comparison, and outcome) format was used to guide our research question: “Are Indo-Caribbean adolescents (P) living in CARICOM nations (I) at an increased risk of suicide (O) compared to other ethnic groups (C) in CARICOM nations?”

## 2. Materials and Methods

### 2.1. Eligibility Criteria

Studies included for review required the following criteria to be considered for inclusion: (a) focus on any sole country or combination of countries within Guyana, Suriname and Trinidad and Tobago, (b) quantitative data pertaining to the demographics of study participants, (c) analysis of suicidality, and (d) analysis of adolescent participants in the study.

### 2.2. Information Sources and Search Strategy

This review was completed in accordance with the WHO rapid review guide [8]. The protocol was registered with PROSPERO (CRD42023417494). The literature search was carried out from database inception to 17 February 2024 via database searches, with the target literature including studies pertaining to and analyzing suicidality in the countries of Guyana, Suriname, and Trinidad and Tobago. Databases considered included PubMed and APA PsycInfo. Database suitability was determined via results return following entry of a probing search term—“guyana suicide”. Following a positive result return, suitability was established for the literature search utilizing these databases (File S1).

Prior to search execution, the filters were set on the databases to include full text and peer-reviewed literature. The filters on PubMed were set for “full text” and “free full text”. It should be noted that APA PsycInfo was accessed via EBSCOhost. Filters were set to limit the search to “full text”, “scholarly (peer-reviewed) journals”, and “open access”. The default search mode was “Boolean/phrase” and additional mode options included “find all my search terms” and “find any of my search terms”. If the default search mode yielded no results using a search term, the search mode was changed accordingly until results were obtained, if any.

When beginning searches on a specific topic such as a country or ethnicity grouping, the searches focused on Guyana included the “guyana suicide” probing term as well as additional search terms. These searches maintained “guyana suicide” followed by an “AND” Boolean operator to be used in further searches, with separate searches including “(youth OR adolescent)”, “(support OR bereavement OR postvention)”, “(victims OR survivors)”, “(prevention OR reduction)”, and “(stigma OR taboo)” following the “AND”, to search for literature including key terms. The final Guyana-focused search was “guyana mental health resources” to conclude the Guyana-based literature search.

Subsequent searches sought to include the Indo-Caribbean context as a whole with regard to adolescent suicide. In order to reduce search result ambiguity due to punctuation, the initial probing search term was “indo-caribbean OR indo caribbean suicide”. Four following searches were composed of the probing term nested in parentheses, followed by an “AND” operator with “(youth or adolescent)”, “(prevalence)”, “(stigma OR taboo)”, and “(postvention OR bereavement)”. The final Indo-Caribbean-focused search was “indo-caribbean OR indo caribbean mental health”.

The final two searches sought literature focused on the context of youth suicide and bereavement in the countries of Suriname and Trinidad and Tobago. The search terms were “(suriname suicide) AND (youth OR bereavement)” and “(trinidad suicide) AND (youth OR bereavement)”.

### 2.3. Study Selection

Initial literature screening consisted of analysis of the title and provided abstracts after filtering duplicate records from PubMed and APA PsycInfo. One author independently selected studies, then both authors assessed the methodological quality of the eligible studies together. Disagreements on the eligibility of studies were resolved through discussion between the two authors and eventual consensus.

Records containing abstracts focusing on the Jonestown massacre were excluded, since the majority of the victims were American citizens, which would be unrepresentative of general Guyanese demographics [9]. Also excluded via screening were studies not focusing on Guyana, Suriname, or Trinidad and Tobago, since the aforementioned countries are the focus of the current study. Lastly, abstracts not mentioning suicidality were also excluded during screening.

Assessment for inclusion eligibility was carried out via analysis of full-text contents. Literature reviews were excluded. Studies that did not feature an analysis or mention of suicidality were excluded. The WHO defines the adolescent age range between 10 and 19 years, and studies featuring participants within this range were considered for inclusion [10]. Studies that did not numerically quantify the studied sample/population size or participant age ranges, gender, and ethnicity were not eligible for inclusion. In order to select literature with the most broad and inclusive samples/populations that did not include an Indo-descendant ethnicity grouping, studies focused on a sole ethnic group, and those only including one gender were not selected for review. By keeping the samples in the selected literature broad and inclusive, it would be possible to visualize and highlight the differences and nuances in the relationships of different genders and ethnicities with regard to the risk factors of suicidal behavior.

### 2.4. Quality Appraisal

Both authors assessed the methodological quality of the eligible studies. Consideration for inclusion was based on their relevance to the subject of this review. All of the studies contained samples best representative of the multi-ethnic nature of the countries of focus featuring adolescent participants. The study samples comprised at least 20 individuals. All of the studies contained stratified data respective of their participants’ demographics (e.g., gender, ethnicity). Studies with broad and inclusive study samples were selected to minimize any bias of findings toward any specific gender, age, or ethnic group. Most of the participants in the samples in the included studies were based on voluntary participation, with the other studies consisting of a secondary data analysis from official registers (e.g., hospitals and law enforcement) to obtain their samples. Not all studies included precision measurements such as confidence intervals or *p*-values.

### 2.5. Data Extraction and Synthesis

Both authors performed data extraction for the studies found to be eligible for inclusion after screening their methodological quality. Extraction of data from the identified literature required review and analysis of the full-text contents, with collected data pertinent to the research objective, which was adolescent suicidality in Guyana, Suriname, and Trinidad and Tobago. Data to be included consisted of (1) author information, (2) publication year, (3) article title, (4) study design and methodology, (5) country of focus, (6) adolescent participant age ranges, (7) participant genders, (8) participant ethnicities, (9) sources of data, (10) primary outcome(s) of study, and (11) key findings from the literature.

The countries of Guyana, Suriname, and Trinidad and Tobago are each ethnically diverse, with significant populations hailing from different parts of the world, as well as local indigenous and assimilated ethnicities. The countries officially refer to these groups with unique identifiers. Ethnicities provided in the studies are categorized as “Afro-Caribbean” (includes “Afro”, “African”, “Black”, “Creole”), “Amerindian” (includes “Amerindian”, “Indiaan”), “Indo-Caribbean” (includes “East Indian”, “Hindustan”, “Hindustani”, “Indian”, “Indo”), “Multi-racial” (includes “Mixed”, “Multiracial”), and “Other” (as per “Other” category in studies where ethnicity is unlisted, unknown or unclear). Other significant ethnicities included in the studies are “Javanese” and “Portuguese”.

## 3. Results

### 3.1. Summary of Included Literature

**Figure 1:**
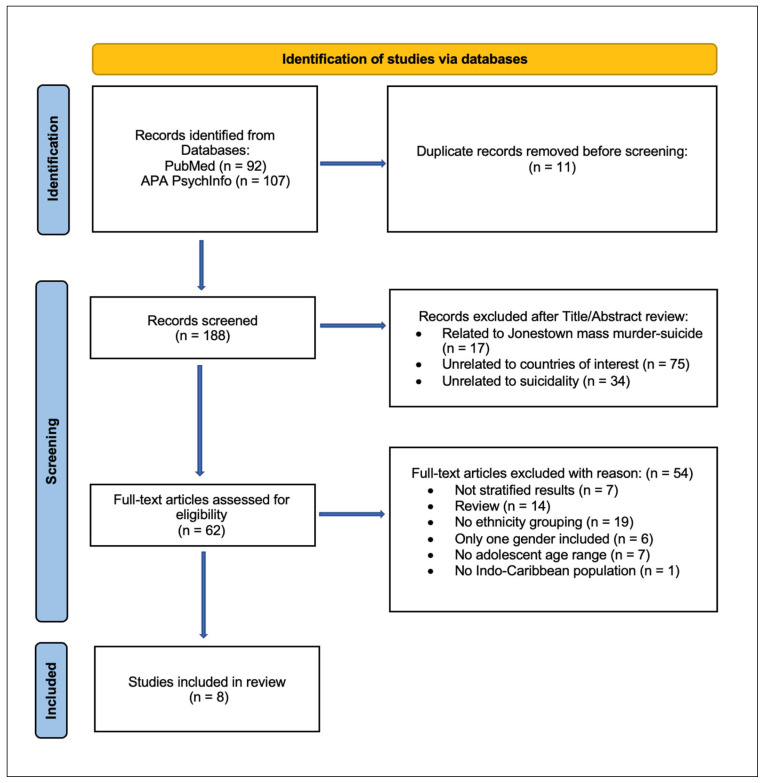
Study flow based on the 2020 flow diagram for new systematic review, which included a search of databases and registers only [11].

A search of two databases resulted in a total of 199 papers, which included 11 duplicates. The literature selection process was documented in a flow diagram in accordance with the Preferred Reporting Items for Systematic Reviews and Meta-Analyses (PRISMA) guidelines (File S2) for systematic reviews and meta-analyses [11]. Figure 1 presents the flowchart of the screening and selection of the literature. Following the filtering of duplicates, 188 papers were screened through reading of the titles and abstracts alone, after which the remaining 62 papers were assessed for eligibility by analysis of full-text content, resulting in a total of 8 papers to be included for review, encompassing 6581 individuals.

Table 1 provides an overview of the data extracted from the included literature. Each study pertained to a singular country, with five studies focused on Guyana [12,13,14,15,16], two studies focused on Trinidad and Tobago [17,18], and one study focused on Suriname [19]. Study designs varied, including mixed-method (*n* = 3) and quantitative *(n =* 4) studies. The three mixed-method studies were published within the past five years (since 2018) and utilized cross-sectional methodology. Of the quantitative studies, three were cross-sectional and one was a secondary data analysis, with the two oldest studies published in 2005 and 2006 and the two most recent published within the last 10 years (since 2013).

Regarding the age ranges of the study participants, studies included adolescent ages within age groups of slightly older or younger than the 10- to 19-year age boundaries (*n* = 3), broadly mentioned adolescents as a focus of study without defining an age range *(n* = 2), and included age ranges within the WHO-based adolescent age boundaries *(n* = 2). The quantitative study by Toussaint et al. does not list its age ranges, instead mentioning the highest three education levels of the adolescent participants, with “Form 5” grade level in Trinidad and Tobago presented as an equivalent to United States Grade 12, with a sample mean age of 18.14 years, within the WHO-based age range [18]. Although the included mixed-method study by Johnson does not provide participant age ranges, it is included since its data are sourced from the Welfare Office of the Police Department in Guyana, which operates a suicide hotline primarily serving adolescents who have at one point considered or attempted suicide, and was sought by the researcher specifically for the collection of data focused on adolescents [13]. The qualitative study from Guyana [16] examined youth between the ages of 10 and 18 years (*n* = 5) with fatal suicide attempts. Gender and ethnicity data were present in all included studies.

### 3.2. Guyana

The majority of the included studies were conducted in Guyana, employing mixed-method (*n* = 3), qualitative (*n* = 1), and quantitative (*n* = 1) methodologies. The mixed-method and quantitative studies utilized a cross-sectional design. All of the studies considered ethnicities of the adolescent participants and records of adolescents, as well as gender. In total, the studies accounted for 224 adolescent individuals.

The most recent study was performed in 2023 [16] and found that loss of lives to suicide in Guyana are often due to reasons such as interpersonal conflict, previous trauma, and physical/mental health status, and also for reasons unknown to family members of individuals who died by suicide. The authors also note that the two means of suicide observed in the study are pesticide poisoning and hanging. The majority of the individuals lost to suicide in this study were male (*n* = 14). Also noteworthy is the ethnicities of those lost to suicide in this study, with the majority of individuals (*n* = 16) being Indo-Caribbean.

The study performed by Denton [15] found that that the majority of non-fatal suicide attempts were female (54.5%). A major risk factor for suicidality mentioned in this study is the experience of previous trauma in the lives of youth, in which the number of traumatic events was positively associated with age (r = 0.32, *p* = 0.02), the presence of anxiety (r = 0.30, *p* = 0.03), rate of suicidal ideation (r = 0.46, *p* = 0.01), and number of suicide attempts (r = 0.56, *p* = 0.01).

The mixed-method study by Johnson [13] found that the individuals most susceptible to suicidal behavior were females (55%), a finding consistent with Denton’s study [15], as well as individuals of Indo-Caribbean ethnicity. Also worth noting is that Johnson’s study cites household conflicts (e.g., domestic violence, intimate partner violence) and the rejection faced by youth after coming out as gay or lesbian as contributing factors toward suicidal behavior. Domestic violence is defined by the United Nations as behavior that is used in a relationship to gain or sustain power over an intimate partner, a child, or any other household member/relative [20]. Intimate partner violence is defined by the WHO as behavior by a relationship partner that causes physical, sexual, or emotional harm, often through aggressive, coercive, or controlling behaviors within a romantic relationship toward another relationship partner [21].

Indo-Caribbean ethnicity was also identified as the ethnic group most susceptible to suicidal behavior in a study conducted by Arora et al. [14] that sought to identify risk factors for suicidal behavior in adolescents. Other risk factors found by the authors included the transition from late adolescence to adulthood and high expectations from parents, teachers, and religious leaders, as well as the expectation by these figures of youth adherence to cultural norms.

A study conducted by Arora and Persaud [12] noted that the fear of gossip is a major barrier in mental health help seeking in adolescents, stemming from the desire by individuals and institutions to preserve social reputation. The authors of the study also mention that there is a limited awareness and inaccessibility of mental health resources, a notable obstacle preventing youths from seeking aid.

### 3.3. Trinidad and Tobago

Two of the studies included in the review were conducted in Trinidad and Tobago, both employing quantitative methodology and cross-sectional design. As with the Guyana studies, the Trinidad and Tobago studies also regarded ethnicity and gender of the adolescent participants, accounting for 6253 individuals.

The most recent study, conducted by Toussaint et al. [18], found that the individuals at highest risk of suicidal ideation and planning were females (57%), a similar finding to the Guyana studies. Also at high risk were multi-racial individuals compared to Indo-Caribbeans. A notable protective factor against suicide in this study appeared to be religious participation, wherein a higher rate of participation was associated with lower likelihood of suicidal behavior, demonstrating an inverse relationship between the two. The authors also note that compared to Indo-Caribbeans, Afro-Caribbeans are more likely to be treated for suicide attempts. Also noteworthy from the study is an inverse relationship between level of education completed and suicide planning.

A 2005 study by Ali and Maharajh [17] identifies females as having higher rates of suicide attempts, consistent with Toussaint et al., and also identifies Indo-Caribbean as the ethnicity with higher rates of suicide attempts, which differs from Toussaint et al. [18], who placed multi-racial individuals at higher risk. The findings of this study correlate the variance of family structures and presence alcohol abuse within households with risk factors of suicidal behavior. A protective factor identified in this study is higher frequency of religious participation, a protective factor also mentioned in the study by Toussaint et al., potentially illustrating the role religion plays in Trinidad and Tobago. With regard to education, the findings of this study suggested no significant relationship between suicidal behavior and the type of school attended by youth.

### 3.4. Suriname

Graafsma et al. [19] employed quantitative methodology to examine data from 2000 to 2004. Consistent with the studies conducted in Guyana and Trinidad and Tobago, ethnicity and gender of the participants was regarded, accounting for 104 individuals. The ethnic group found by the author to be most susceptible to suicidal behavior was Indo-Caribbean. This study notes that fatal suicide attempts are more prevalent in males (75%), and non-fatal suicide attempts are more common in females (51%). Graafsma et al. found that the two leading methods of suicide are pesticide poisoning and hanging. The authors of this study also highlighted the demographics of the city of Nickerie, the second-most populated city in the country. At the time of this study’s publication, Indo-Caribbeans comprised the majority of the fatal and non-fatal suicide attempts (80% and 83%, respectively) in Nickerie.

## 4. Discussion

When investigating an issue as complex and multi-layered as suicidal behavior, it is crucial to understand the role that individual, family, and community risk factors play in the development of suicidal ideation and attempts in order to guide the appropriate responses to reduce and prevent the development of these behaviors as closely to the source as possible [1]. Knowledge of these specific risk factors and the way that they affect adolescents with respect to the culture in which they live would prove useful to shape competent suicide prevention efforts.

### 4.1. Gender Differences

Upon examination of the literature findings in Guyana, Suriname, and Trinidad and Tobago, it has been observed that adolescent females are more susceptible to suicidal behavior than males. Data recorded by the United Nations International Children’s Emergency Fund (UNICEF) [22] shows that among female adolescents aged 15–19, self-harm is the leading cause of mortality in Guyana, second-leading cause in Suriname, and fourth-leading in Trinidad and Tobago. In males of the same age group, self-harm is the leading cause of death in Guyana, third leading in Trinidad and Tobago, and fourth-leading in Suriname. The same data also place depressive disorders as the leading cause of years of healthy life lost due to disability (YLDs) among females in this age group in Guyana, Suriname, and Trinidad and Tobago. Among males, it is the leading cause of YLDs in Suriname and Trinidad and Tobago and the second-leading cause in Guyana. In each of the three countries, the data also show that the use of alcohol and tobacco is more common among males than females, which likely indicates a social environment in which substance use disorders are prevalent among youth.

The influence of the social environment itself will have different effects on individuals of different genders. As found in Denton’s study [15], major risk factors in young Guyanese females include previous traumatic events and anxiety symptoms. Johnson’s 2019 study [13] mentioned the presence of intimate partner violence and domestic violence as a reason for adolescent females to seek counseling. Both of these Guyana-centered studies show the prevalence of traumatic experiences and intimate partner violence, which appear to be major contributing factors toward suicidality in adolescent females. A 2010 cross-sectional study exploring the prevalence of abuse toward women in Trinidad and Tobago (Maharaj et al., 2010) similarly notes that physical and sexual abuse is commonly reported, along with anxiety and post-traumatic stress disorder (PTSD). This study contains a small subset of the sample consisting of 12 individuals aged 18–19 years, which may not accurately reflect the experiences of younger women in a broader societal context. It is worth noting that the ethnicity most represented among the sample was Indo-Caribbean, a finding similar to Johnson’s 2019 study, where Indo-Caribbean adolescent females made up the majority of the individuals seeking aid.

A 2021 study [23] conducted in Guyana on the self-reported health outcomes of females aged 15–64 years associates intimate partner violence with higher odds of suicidal ideation (95% CI: 2.48–6.76; *p* < 0.001), as well as higher odds of poorer reported overall health (95% CI: 1.00–2.24; *p* < 0.05) in women during pregnancy. This study associates increased likelihood of intimate partner violence with factors such as controlling partner behavior, being unmarried, previous experience of physical violence during childhood, and alcohol abuse by the relationship partner. Although Miller and Contreras-Urbina’s study does not classify participants based on ethnic groups, it does demonstrate a gap in knowledge of these issues along different cultural lines, which may prove useful in the development of culturally competent approaches to curb domestic violence and its effects on young women.

The authors of a 2023 study from Guyana [16] indicate that domestic abuse is a major contributing factor to fatal suicide attempts in women, a finding consistent with Denton [15] and Johnson [13], who attribute this factor to non-fatal suicide attempts among youth in their respective studies. The findings of the previous studies are in line with a study from Shaw et al., and also found that the majority of individuals lost to suicide are males, although it is unclear how many of the risk factors affect the few adolescent males and females mentioned. The lead author of the study at the time of publication was a clinical psychology PhD candidate as well as a registered provisional psychologist. The author utilized a psychological autopsy approach to examine the background of the lives of the individuals lost to suicide, which was accomplished by interviewing the living relatives and acquaintances to obtain information about the lives of the focal individuals who were lost to suicide. Future studies that employ the psychological autopsy method of this study may also prove useful in examining root causes of suicidality in youth contexts to further understand the risk factors affecting them the most.

In adolescent males, a 2003 study [24] exploring adolescent health in the Caribbean, including Guyana, mentions the experience of feelings of rage among youth, with males reporting rage significantly more than females. Rage is defined by the study as the feeling of anger to the point one “could kill someone”. The rage is found to be experienced after traumatic events such as the witnessing of suicide and past experiences of abuse. The authors also note that young Caribbean males who report feelings of rage are more likely than females to demonstrate violent tendencies toward other people. While this study did not examine the youth in ethnic contexts, it does represent the pervasiveness of rage in Caribbean contexts. Further research into these feelings of rage among youth in Suriname and Trinidad and Tobago together with Guyana can be used to determine whether an ethnic/cultural component in the management of rage also exists in those countries’ populations, and if contributes at all to risk factors of suicidality and violent tendencies among youth.

Research findings by Blum et al. (Blum et al. 2003) report that rage is a common finding among young Caribbean males, which is a likely catalyst for violence in romantic/cohabitating relationships. Such findings could also explain the occurrence of homicide–suicide (H-S) in the Caribbean. A 2012 peer-reviewed commentary article by Emmanuel and Campbell [25] attributes the motives of H-S as intimate partner violence, often with the perpetrator as male and the victim as female. Among the countries mentioned in this article are Guyana and Trinidad and Tobago, with Suriname absent. While the authors note that these occurrences are rare and that research on the subject is scarce, there is a noticeable need for greater awareness of H-S and the prevalence of mortalities associated with H-S. It may be possible that within the recorded suicide and homicide statistics in these countries, there may be individuals who are perpetrators of homicide and victims of homicide. A greater understanding of this phenomenon and its prevalence would aid in the swift identification of risk factors and prompt action toward individuals who are potential perpetrators or victims before such tragic events unfold, although further research is needed to understand what other factors contribute to H-S as well as its relative frequency.

### 4.2. Family Structure and Relationships

Multiple factors such as family structure and the relationship between youth and their families can have an effect on the manifestation of suicidal behavior. When examining suicide attempts in adolescents, the 2005 study by Ali and Maharajh conducted in Trinidad and Tobago [17] shows a trend when examining the relationship with family structures. The odds of suicidal behavior in relation to family structure are as follows (*p* < 0.01): intact families (6.1%), living with relatives (8.3%), one-parent families (9.6%), reconstituted families (14.6%), and single step-parent families (16.7%). The presence of alcohol in the household also has a relationship with suicidality, with significantly higher rates (*p* < 0.001) of both suicidal ideation and suicide attempts in households with alcohol abuse present compared to households where alcohol abuse is absent.

It is also necessary to understand the risk factors affecting youth in alternative living arrangements, such as the participants in Denton’s study [15], which focused on Guyanese youth living in orphanages. Although the study may not capture the experience of adolescents in Guyana as a whole, it did examine a unique group of young people whose lived experiences differ from youth living with family members, especially during the formative years of their lives. Future research on youth and suicidality in relation to living arrangements could further include youth living in orphanages in Guyana, Suriname, and Trinidad and Tobago to further guide developments in suicide prevention.

In the context of youth with suicidal feelings and their family units, there is a noticeable barrier to conversations about suicide or suicidal ideation. A 2019 study by Arora and Persaud [12] sought input from school-going youth in Guyana, and found that individuals will often avoid the topic of suicide with their parents for the sake of avoiding shame in the household, which is due to a prevalent social stigma with regard to suicidality. This element of youth refraining from speaking to family members is echoed in a 2017 focus group study conducted in Guyana [5], in which youth commonly cite lack of family support or corporal punishment as a barrier to speaking out, as mentioned by the nurses who attend to suicidal youth. A dismissive or hostile reaction from parents presents a major risk factor of suicidal ideation, as youth concerns regarding suicidal ideation are not addressed appropriately. Such unaddressed concerns may only exacerbate ideations, potentially leading to subsequent attempts.

In the case of Suriname, the perception of parental corporal punishment toward youth in Indo-Caribbean families was examined in a 2018 study conducted in four Surinamese cities [26]. The authors note the prevalence of corporal punishment in Suriname, as mentioned by the study participants, who express that it is a common occurrence in their communities. Although the study was not specifically focused on suicidal behavior, the youth participants and caregiver participants specifically mention suicide as a consequence of corporal punishment. Since corporal punishment is a sensitive topic, the authors note that the focus group methodology may influence participants to underreport their experiences due to confidentiality concerns. Future studies exploring this topic may be conducted using single-participant interviews to increase the participants comfort in confidentiality, potentially leading to more candid and descriptive responses on delicate subjects such as corporal punishment and suicide.

Other familial attitudes are explored in a 2020 cross-sectional study (Arora et al., 2020), which often stem from cultural and religious expectations that Guyanese parents maintain for their adolescent children. Parents expect youth to behave and present themselves in certain ways, which becomes a source of friction between family members in the form of dismissing or invalidating youth who express suicidal feelings. The strict cultural adherences appear to be a point of conflict with the multi-ethnic nature of the Caribbean populations, a structure that is likely also present in the similarly multi-ethnic populations of Trinidad and Tobago and Suriname. An example of the dismissal by parents provided in this study is the opposition to their children’s pursuit of inter-ethnic romantic relationships, which is a major source of stress for the youth and a notable risk factor for suicidal behavior.

A 2018 study utilizing data from the Global School-Based Student Health Survey (GSHS) [27] examined the relationship between parental involvement and mental health, including data from Trinidad and Tobago. The study found that higher levels of parental involvement are inversely related with suicide attempts among adolescents (*p* < 0.001), as well as lower rates of suicidal ideation (*p* < 0.001), anxiety (*p* < 0.001), and feelings of loneliness (*p* < 0.001). Similar studies could be conducted in Guyana and Suriname that analyze the relationship between parental involvement and mental health of adolescents in order to gain an understanding that could also include an ethnic component to visualize if parental involvement toward youth has different effects on mental health within an ethnic/cultural context. Further studies could also seek to identify household risk factors of adolescent suicide unique to Indo-Caribbean groups, as well as those unique to other ethnicities.

### 4.3. Social and Societal Pressures

The social environment of youth in Guyana, Suriname, and Trinidad and Tobago can be highly influential in the manifestation and management of suicidal behavior, ranging from ethnic/cultural aspects to social pressures in the life experiences of youth. A palpable social stigma pertaining to suicidality extends deep into society, as observed by student participants in Arora and Persaud’s 2019 study from Guyana [12]. The authors of this study note that institutions will often avoid discussing suicide in order to prevent gossip from ruining their prestige or reputation by association with suicide. The participants mention a case in which a fellow classmate lost their life to suicide, an incident that the school largely dismissed publicly to avoid damaging its reputation. It is also worth noting that although this finding may be coincidental, the majority of the voluntary participants in Arora and Persaud’s study (*n* = 35) are of “East Indian” ethnicity. According to Arora et al. (Arora et al. 2020), the “East Indian” ethnicity was identified with the highest risk of suicidality by the youth interviewees, due to the prevailing cultural attitude that East Indians endure and internalize stress, leading them to feel overwhelmed, a likely contributing factor to the general stigma regarding suicide. Further cross-sectional studies in Guyana, Suriname, and Trinidad and Tobago may help to identify and clarify factors that contribute to the stigmatization of suicide to better shape future prevention strategies.

Another risk factor likely influencing social pressures is geographical location. The 2006 study from Suriname [19] mentions the city of Nickerie, which is the second-most populous in the country. It is comprised of an ethnically diverse population containing an Indo-Caribbean majority (70%). It is also directly on the border with Guyana, a location that likely sees a flow of people between the two countries. Another area for future study would be the effect of geographical location on the mental health of different population centers of the countries with relation to the proximity to neighboring countries. This may further clarify whether the migration of people between these two countries has a relationship to the mental health burdens faced by Guyana and Suriname, which happen to have the highest and second-highest suicide rates in the Americas [3].

It is also important to consider and understand the relationship between culture and mental health outcomes. While not directly focused on youth living in the countries of interest of this review, a cross-sectional study [28] conducted in the United States examined the mental health outcomes of older South Asian adult immigrants of various origins. The participants with origins in Guyana and Trinidad and Tobago (Indo-Caribbean South Asian) experienced the highest prevalence rates in the outcomes of focus, which were loneliness (48%), mild/moderate depression (16%), and emotional distress (52%), and the second-highest rate in difficulties due to depression symptoms (18%). The authors note that there is a disproportionate mental health burden in the Indo-Caribbean grouping. While this study did not include Indo-Surinamese individuals, a similar finding was observed in a 2018 study conducted in the city of Rotterdam in the Netherlands [29]. The focus of this study was non-fatal attempted suicide in females aged 14 to 16 years, with the study sample also stratified according to ethnicity. Among the ethnicities included in the study was “Creole-Surinamese” which aggregates “Surinamese”, “Surinamese/Creole”, and “Surinamese/South Asian” groups into one single classification. It was found that the Creole-Surinamese showed the highest rate (15%) of attempted suicide (*p* ≤ 0.05) when compared to the other ethnic groups in Rotterdam. The Creole-Surinamese findings in the study do not explicitly attribute factors specific to any Surinamese ethnicity, although it likely signals a notable mental health burden present in Suriname that spans across its population. While the social and cultural norms differ in the United States and the Netherlands for different age groups, there is an indication that the South Asian-descended Indo-Caribbean groups may experience a unique mental health burden that follows them upon immigration or that may be exacerbated by immigration. Further studies could examine if there is a cultural factor present in Indo-Caribbean and South Asian immigrant populations abroad that may affect the observed mental health outcomes and non-fatal suicide attempt rates in the aforementioned studies.

Equally important to consider are the social pressures (e.g., peer pressures) observed among youth in Guyana, Suriname, and Trinidad and Tobago. Blum et al. [24] note that substance abuse, namely, of tobacco, alcohol, and cannabis, is prevalent among youth in these countries. The authors also note that substance abuse is more common in youth who experience parental abuse and also witness parental substance abuse and parental mental health issues. Other external factors include rage and knowing a friend or family member who attempted suicide. Maharajh and Konings in a 2005 pilot study conducted in Trinidad and Tobago [30] briefly outlined that cannabis use in particular has seemingly integrated into Caribbean culture. The authors attributed cannabis use to the arrival of indentured servants from India, who used cannabis as a means of adjusting to what was perceived as a hostile environment. The authors further indicate that the use of cannabis became somewhat normalized in Caribbean society to the point where adolescents began to use it themselves. With regard to youth who have been exposed to suicide of loved ones, further research into the bereavement of youth may be useful to identify potential triggers that could arise during grief following a loved one lost to suicide.

While the 2005 pilot study by Maharajh and Konings [30] mainly provided the context to examine the relationship between cannabis use and suicidal behavior and adolescents, a study published in 2008 by Konings et al. [31] is a completed study which follows the premise of the pilot study. The 431 participants were randomly selected from schools in Trinidad and Tobago to obtain a representative sample of the Trinidadian population, and the participants completed questionnaires that investigated cannabis use and psychotic symptoms. The questionnaires were administered in the school and supervised by a teacher. The ages of the participants ranged from 12 years to 21 years, with a mean age of 16 years. The study found that although cannabis overall is not associated with higher rates of psychotic symptoms (β = 0.14, 95% CI: −0.10; 0.37, *p* = 0.25), the age of first cannabis use does have an effect on the manifestation of psychotic symptoms. First use of cannabis at early ages showed greater effects toward psychosis outcomes in youth (F (1, 371) = 3.91, *p* = 0.05), with first use of cannabis below age 14 showing the greatest association with psychotic symptoms in the sample (β = 0.39, 95% CI: 0.04; 0.74, *p* = 0.029). The aforementioned association was not seen in participants above the age of 14. The authors noted that at the time of publication, this was the first study of its kind to analyze the relationship between cannabis use and psychotic experiences in a non-Western country with a multi-ethnic population.

Further studies of similar scope to Konings et al. [31] could also prove useful in other multi-ethnic countries such as Guyana and Suriname, especially since they also have an ethnic plurality comparable to Trinidad and Tobago. It would also be beneficial to examine if other substances are associated with psychotic effects in youth of these countries, to better address any underlying risk factors to suicidal behavior.

### 4.4. Methods of Suicide

The availability of the means of suicide to youth can be considered a risk factor of suicidal behavior in its own right. A 2017 research article [32] briefly mentions a pathway to action in the manifestation of suicidal behavior, referred to as ideation-to-action. This entails a combination of three factors present in an individual, which are psychological pain with sense of hopelessness, the feeling of disconnection, and the means or capacity to attempt. An example of this manifestation is seen in a 2018 focus group study in Suriname [26] in which youth participants mention the case of a 16-year-old girl in Suriname who experienced severe corporal punishment to the point where the adolescent ended her life by use of pesticide.

Pesticide poisoning appears to be a prevalent method of suicide in the Caribbean, as also seen in a 1999 study conducted in Trinidad and Tobago [33], with the pesticide used in the cases of suicide identified as Paraquat (gramoxone) and the ethnicity most vulnerable to poisoning the “East Indian” or Indo-Caribbean group in the country. A 1988 study focused on a rural community in Trinidad and Tobago [34] also shares this finding of the Indo-Caribbean ethnicity being susceptible to suicide via ingestion of Paraquat. It is certainly remarkable that this is a subject of study dating back nearly 30 years from the writing of this review, which underscores the importance of greater research and solution pathways to an observed phenomenon that is costing the lives of individuals.

The Surinamese study by Graafsma et al. [19] found that 75% of all fatal suicide attempts and 49% of all non-fatal suicide attempts were by males, with 35% of the male fatal suicide attempts falling within the age group of 16 to 25 years. Suicide attempts were also found to be more common in youth, with 11% of non-fatal attempts consisting of individuals aged 15 years and under and 40% consisting of individuals between 16 and 25 years being overrepresented among fatal and non-fatal attempts compared to older age brackets. This study examines the documented methods of suicide, and points out that gender differences in method are difficult to ascertain since the numbers are relatively small, although notes that four out of the five women who lost their lives to suicide used pesticide intoxication. The study notes that pesticides were the most prevalent method of suicide in 2004 (55%), followed by hanging (40%). When looking at non-fatal attempts, 48% consisted of non-pesticide intoxication (e.g., chlorine, turpentine, nail polish remover, ammonia, vinegar acid and prescribed drugs), 44% consisted of pesticide intoxication, with the most prevalent pesticide being Paraquat, with other plant pesticides and animal poisons also listed, and 4% of non-fatal attempts involved the use of knives. With the available data, it was determined that 63% of male non-fatal attempts used pesticide intoxication and 75% of female non-fatal attempts used non-pesticide intoxication (*p* < 0.05). Pesticide use was found to be evenly distributed among all age groups. The study found that 80% of fatal suicide attempts and 83% of non-fatal suicide attempts in Nickerie involved individuals of “Hindustani” ethnicity, which the authors point out is not significantly different from the ethnic composition of the district’s population. When compared to Paramaribo, Nickerie was found to have twice the suicide attempt rate, with 78 individuals per year presenting to the hospital for treatment from suicide attempts compared to 379 individuals per year in Paramaribo presenting for treatment, a figure noted by the authors as remarkable. The authors also note that registration of data for suicidal behavior for the country as a whole is lacking, which would suggest that the figures presented for Suriname as a whole are underestimations.

As for Guyana, a literature review published in 2022 [35] also shows that there is literature indicating the presence of suicide by pesticide poisoning in Guyana. The review cites a 1968 study [36] on 36 individuals who attempted suicide in Guyana. A major sociocultural element present in McCandless’ analysis of the “East Indian” demographic of the sample is parental rejection. A notable finding indicates that among the “East Indian” individuals, who also comprised the majority (67%) of attempted suicides in the sample, it is commonly reported that parents would express frustration by telling their children to kill themselves or to ingest pesticide. There is, however, limited knowledge on the exact vulnerability of adolescents utilizing pesticide and if any ethnic groups in Guyana are specifically vulnerable to suicide by this method compared to other methods. In their 2023 study [16], Shaw et al. list pesticide poisoning and hanging as the two methods utilized in the fatal suicide attempts they examined, a finding similar to Graafsma et al. [19], although with a much smaller sample. Further research in the Caribbean may focus on the ease of accessibility of pesticides and other means of committing suicide, as well as if any ethnicities or age groups are uniquely susceptible to suicide via pesticide poisoning.

### 4.5. Protective Factors against Suicide

While Indo-Caribbean youth face various risk factors, an equally important consideration is the presence of protective factors against suicidal behavior. A notable protective factor observed in the reviewed literature is religious practice. The 2015 study by Toussaint et al. [18] sought to examine the relationship between religiousness and suicidality among adolescents and young adults in Trinidad and Tobago. When examining the associations between religion and suicidal behavior, the groups least likely to plan suicide than the non-religiously affiliated individuals were shown by this study to be Catholics, Seventh-Day Adventists, and Pentecostals, with Methodists and Seventh-Day Adventists showing lower odds of suicidal ideation. The groups more likely to be treated for suicide attempts than non-religiously affiliated individuals were shown to be Hindus, Muslims, and members of “other” religious groups, as mentioned by the study. The degree of religious participation also demonstrated a relationship with suicidal behavior. Individuals who perceive themselves to be religious, attend religious institutions, and pray are less likely to contemplate suicide, with the study demonstrating an inverse relationship between the extent of religious participation and the likelihood of suicide attempt in adolescents and young adults. The study does mention, however, that the reading of religious literature is associated with increased suicide planning, while prayer shows a decreased likelihood of being treated for a suicide attempt.

In their 2005 study [17], Ali and Maharajh also noted that religiosity appeared to influence the likelihood of suicidal behavior in that individuals who pray with family and/or frequently attend religious institutions present a lower likelihood of suicidal ideation. Regarding suicide attempts and religious practices, frequency of religious institution attendance presented no significant differences in suicide attempt rate, although praying with family did present a significant difference, with higher suicide attempt rates in individuals who did not pray with family than individuals who prayed with family. This observance may indicate an additive effect of parental involvement in the lives of Trinidadian youth, as Pengpid and Peltzer’s study [27] illustrates an inverse relationship between the level of parental involvement and the likelihood of suicide attempts. Further research regarding the protective factors connected to religiosity may elicit perspectives from religious leaders and counselors who encounter adolescent followers that considered suicide or relatives of individuals who lost their lives suicide. That knowledge can serve to construct prevention efforts that are compatible with the religious practices of the youth experiencing suicidal ideation.

Similarly to religious practices, peer support groups and group activities may prove to show a protective effect as well. Adolescent participants in Arora and Persaud’s 2019 study conducted in Guyana [12] expressed that talking about their feelings with other individuals in the same age group can provide a trusted space for them, as well as a greater sense of understanding among peers. Similarly, the participants in the study by Arora et al. [14] mentioned that involvement in group activities in the community can help free their minds and relieve stress. Examples of such activities include yoga, sports, and study groups. A notable group activity that is also mentioned in the study is the *shakha*, which is a Vedic group study and meditation practice, a finding parallel to the religious protective factors observed in studies by Ali and Maharajh [17] and Toussaint et al. [18] in Trinidad and Tobago. It is important to note the overlap between protective religious factors and the protective effects of parental involvement and peer group settings. This overlap illustrates a positive additive effect of familial and social cohesion, an aspect to consider when planning suicide prevention efforts.

### 4.6. Management of Suicidal Behavior

There is a demonstrated need for improved aid delivery to youth at risk of suicidal behavior, as evidenced by the participants in the studies by Arora and Persaud [12] and Arora et al. [14] in Guyana. Youth in both of these studies expressed a sense of distrust within adults, citing reasons such as confidentiality concerns and uncertainty of who may be a trustworthy adult. Although the sense of distrust is present, there is also a sense of insufficiency of resources. Also cited is the high cost to access a mental health provider combined with the belief that services are rendered unnecessarily and are simply set up to collect payments. A notable finding in the 2019 study by Arora and Persaud [12] is the mention of limited awareness and accessibility of mental health resources, as well as the feeling that the government and non-governmental organizations (NGOs) have the capacity to play a greater role in preventing suicide.

The 2006 study from Suriname [19] also suggests a need for improved aid delivery, notably in rural regions. Nickerie is one such population center that depends on agriculture and has a notable absence of industrial and commercial development, potentially indicating a rural population. Also, possibly owing to its location is the near-absence of mental health resources, which is mainly composed of bi-weekly visits by psychiatrists from Paramaribo, the largest city in Suriname 250 km away. The authors note that at the time of publication, there were no available data on the prevalence of mental disorders in Suriname, which demonstrates a need for further research into the mental health-care needs of the country as a whole, as well as its population centers. Such studies may also be applied to Guyana and Suriname to further understanding of mental health aid delivery in the different types of regions in these countries.

A notable effort examined in the literature to meet those needs is a police department initiative in Guyana, as shown by Johnson’s 2019 study [13]. The police department operates a telephone hotline for adolescents to utilize to seek aid when experiencing suicidal ideation. Along with demographics of the callers, the study also contains discussions from a focus group with the police officers tasked with operating the hotline. Officers reported challenges including lack of funding for the operation and inadequate training, leaving them underprepared to aid the callers appropriately. There is also the effect of officers reporting distress when listening to stories, as well as internalizing the situations they hear. In terms of prevention efforts, the author recommends greater evidence-based training for officers, increased support programs available to officers, research into the perceptions of male providers, and increased focus on Indo-Caribbean females. These suggestions by Johnson may prove to be a vital area of research, since it would guide the practices of specific suicide prevention efforts such as those related to hotlines. Evidence-based practices would prove fruitful in other prevention methods as well, and the effectiveness of those other methods is an area to further explore as well.

Another important factor to note is presented by the WHO [1] regarding the influence of the laws regarding suicide in some countries, namely, that it may lead to underreporting and misclassifications of suicide-related events. The legality of suicide in the countries mentioned is highlighted in a 2016 peer-reviewed article [37] that examines the specific laws and statutes covering suicide across many countries. Suicide attempt is illegal in Guyana, although the article authors mention that the judicial system will not sentence suicide attempters to jail, but local law enforcement will still arrest the attempters and subsequently release them. In Suriname, abetting suicide is illegal, and in Trinidad and Tobago, the status is unclear, since the laws protecting individuals from harm do not mention suicide. The social stigma attached to suicide may be due in part to its legality, which may explain why individuals and institutions refrain from engaging conversations on the topic of suicide. It also may explain the reluctance of individuals to share more of their particular situations when seeking help, as seen in some of the clients mentioned in Johnson’s study [13]. Further research may include greater understanding of the effects of legislation pertaining to suicide on the materialization of suicidal behaviors.

## 5. Limitations

Systematic reviews provide objective assessment of the scientific evidence and attempts to minimize bias by utilizing a methodological approach. This review utilized literature from only two databases, PubMed and APA PsycInfo, which leaves the potential for unreviewed literature that may or may not provide more relevant information regarding the topics discussed above. Also, as in any review, our findings may have been influenced by publication bias. There is also no analysis of the gray literature included in this review, which does not account for information from non-peer-reviewed sources. Systematic reviews provide objective assessment of the scientific evidence and attempts to minimize bias by utilizing a methodological approach. Another methodological weakness of this review is the small sample, which could increase the risk of false-negative results resulting from studies with conflicting results.

## 6. Conclusions

This review was focused on three Caribbean countries with high suicide rates, which also contain significant Indo-Caribbean populations. The results reveal a cultural stigma present in Indo-Caribbean populations that while scantly documented, still shows a role in the social dynamics and subsequent conversations about suicide. Despite the stigma attached to suicide, there is a demonstrated interest within these countries to make efforts to curb suicidal behavior among youth. The studies identified in the review provide greater clarity on cultural perceptions of suicide and the needs of youth at risk of suicidal behavior. However, greater research is needed in areas such as the effects of laws on suicide, the bereavement of families and individuals who have lost loved ones to suicide, greater clarity of sociocultural contexts unique to the individual countries, as well as models of care and suicide prevention methods that are culturally compatible with the Indo-Caribbean populations. Another important consideration for future research includes factors related to the location of vulnerable populations such as the effects of geographic locations of people within a country on mental health outcomes. Also related to location are migration patterns and the mental health burdens experienced by migrant populations of Indo-Caribbeans traveling to other countries. A greater knowledge of these topics may subsequently assist the implementation of future youth suicide prevention methods.

## Figures and Tables

**Table 1:** Study characteristics of articles reporting on suicidal ideation among Indo-Caribbean adolescents living in the Caribbean Community (*n* ≥ 20 participants).

Author(s), Year	Study Design and Method	Age, Mean (SD), y	Gender Differences (Female/Male)	Ethnicity	Source(s) of Data	Key Findings
Guyana						
Arora and Persaud (2019) [12]	Mixed-method; cross-sectional	14–17 (*n* = 40) Mean age: 15.15 SD: 1.00	M: (*n* = 17) F: (*n* = 23)	Afro-Caribbean: (*n* = 2) Indo-Caribbean: (*n* = 35) Multi-racial: (*n* = 3)	Single-participant interviews	Fear of gossip and confidentiality concerns limit youth discussion of suicide. Preservation of social reputation prevents suicidality discussions. Limited awareness and inaccessibility of mental health resources.
Arora et al. (2020) [14]	Mixed-method; cross-sectional	12–17 (*n* = 40) Mean age: 15.15 SD: 1.00	M: (*n* = 17) F: (*n* = 23)	Afro-Caribbean: (*n* = 2) Indo-Caribbean: (*n* = 35) Multi-racial: (*n* = 3)	Single-participant interviews	Indo-Caribbean ethnicity identified as most susceptible to suicidal behavior. Transition from late adolescence to adulthood is major contributing factor to suicidality. High expectations by family, educators and cultural/religious institutions pose suicidality risk.
Denton (2021) [15]	Quantitative; cross-sectional	8–17 (*n* = 50) Mean age: 13.20 SD: 2.35	M: (*n* = 13); (*n* = 4) * F: (*n* = 12); (*n* = 21) *	Afro-Caribbean: (*n* = 9); (*n* = 8) * Amerindian: (*n* = 3); (*n* = 1) * Indo-Caribbean: (*n* = 4); (*n* = 6) * Portuguese: (*n* = 1); (*n* = 1) * Multi-racial: (*n* = 6); (*n* = 8) * Other: (*n* = 2); (*n* = 1) *	DSM-V Level 1 Cross-Cutting Clinical Tool Salimetrics^®^ cortisol enzyme immunoassay kit	Previous trauma experienced by youth positively correlated with age, anxiety symptoms and suicidal behavior. Suicide attempters more likely to be female, with higher rates of psychiatric symptoms. Dysregulated cortisol shows a marginal risk as to whether or not youth attempt suicide.
Johnson (2019) [13]	Mixed-method; cross-sectional	Unspecified Mean age: Unspecified SD: Unspecified	M: (*n* = 33) F: (*n* = 41)	Afro-Caribbean: (*n* = 15) Amerindian: (*n* = 2) Indo-Caribbean: (*n* = 38) Multi-racial: (*n* = 2)	Single-participant interviews Welfare Office of the Police Department	Majority of adolescent callers identified as Indo-Caribbean in ethnicity and female. Individuals experiencing rejection after coming out as gay or lesbian comprise a quarter of calls. Household risk factors involve conflict with domestic partners or family members.
Shaw et al. (2023) [16]	Qualitative	<18 (*n* = 5) Mean age: Unspecified SD: Unspecified	M: (*n* = 14) F: (*n* = 6)	Indo-Caribbean: (*n* = 16) Multi-racial: (*n* = 4)	Psychological autopsy interviews	Fatal suicide attempts due to interpersonal conflict can be related to disputes with family or spousal relationships. Fatal suicide attempts due to childhood trauma can be related to exposure to suicide and child abuse. Fatal suicide attempts due to health concerns can be related to physical/mental illness, substance abuse and self-harm. Fatal suicide attempts also occur in individuals with no identifiable reason apparent to relatives.
Suriname						
Graafsma et al. (2006) [19]	Quantitative; secondary data analysis	≤15: (*n* = 0); (*n* = 9) ** 16–25: (*n* = 7); (*n* = 34) ** Mean age: Unspecified SD: Unspecified	M: (*n* = 15); (*n* = 41) ** F: (*n* = 5); (*n* = 43) **	Afro-Caribbean: (*n* = 1); (*n* = 2) ** Amerindian: (*n* = 0); (*n* = 1) ** Indo-Caribbean: (*n* = 16); (*n* = 70) ** Javanese: (*n* = 2); (*n* = 5) ** Multi-racial: (*n* = 1); (*n* = 6) **	Welzijns Instituut Nickerie Nickerie Police Department Paramaribo Central Bureau of Statistics Paramaribo Bureau of Public Health Care Academic Hospital Paramaribo	75% of all fatal suicide attempts were males, and 51% of non-fatal attempts were females. Non-fatal attempts are more common among youth (15 and under and the 16–25 age brackets combined) compared to older ages. Leading method of suicide in Suriname is pesticide poisoning, followed by hanging. Indo-Caribbean ethnicity most susceptible to suicide in Nickerie and Paramaribo, where Indo-Caribbeans are more populous.
Trinidad and Tobago						
Ali and Maharajh (2005) [17]	Quantitative; cross-sectional	14–20 (*n* = 1810) Mean age: 16.03 SD: 1.13	M: (*n* = 730) F: (*n* = 1078)	Afro-Caribbean: (*n* = 609) Indo-Caribbean: (*n* = 722) Multi-racial: (*n* = 431) Other: (*n* = 431)	Suicidal Ideation Questionnaire (SIQ)	Individuals of female gender and those of Indo-Caribbean ethnicity exhibit higher rates of suicide attempts. Decreased rates of suicide attempts in individuals who frequently attend religious institutions or pray with family. Presence of alcohol in the household and varying family structures influence suicidality in youth. No significant relationship between type of school attended and suicidal behavior.
Toussaint et al. (2015) [18]	Quantitative; cross-sectional	Unspecified Mean age: 18.14 SD: 1.16	M: (*n* = 1911) F: (*n* = 2534)	Afro-Caribbean: (*n* = 1406) Indo-Caribbean: (*n* = 1503) Multi-racial: (*n* = 1477)	TREND Survey	Suicidal ideation rates higher among multi-racial individuals compared to Indo-Caribbean individuals. Afro-Caribbean individuals more likely to be treated for suicide attempts compared to Indo-Caribbean individuals. Higher suicidal ideation and planning rates among females. Higher education levels associated with lower rate of suicidal planning. Frequency of religious participation and prayer inversely proportional to rate of suicide attempts.

* Left-side values denote participants with no previous suicide attempts, right-side values denote participants with previous suicide attempts. ** Left-side values denote recorded absolute fatal suicide attempts, right-side values denote recorded non-fatal suicide attempts. Abbreviations: DSM-V = Diagnostic and Statistical Manual of Mental Disorders, Fifth Edition, F = female, M = male, *n* = number of study participants, SD = standard deviation, SIQ = Suicidal Ideation Questionnaire, TREND = Trend Research Empowering National Development, y = years.

## Data Availability

The authors confirm that the data supporting the findings of this study are available within the article.

## Supplementary Materials

The following supporting information can be downloaded at https://www.mdpi.com/article/10.3390/ijerph21060801/s1. File S1: Search Strategy; File S2: PRISMA 2020 checklist.
